# Phylogenetic and Functional Metagenomic Profiling for Assessing Microbial Biodiversity in Environmental Monitoring

**DOI:** 10.1371/journal.pone.0043630

**Published:** 2012-08-27

**Authors:** Veljo Kisand, Angelica Valente, Armin Lahm, Gerard Tanet, Teresa Lettieri

**Affiliations:** 1 University of Tartu, Institute of Technology, Tartu, Estonia; 2 European Commission - Joint Research Centre, Institute for Environment and Sustainability, Ispra (VA), Italy; 3 Bioinformatics Project Support, Roma, Italy; University of Birmingham, United Kingdom

## Abstract

Decisions guiding environmental management need to be based on a broad and comprehensive understanding of the biodiversity and functional capability within ecosystems. Microbes are of particular importance since they drive biogeochemical cycles, being both producers and decomposers. Their quick and direct responses to changes in environmental conditions modulate the ecosystem accordingly, thus providing a sensitive readout. Here we have used direct sequencing of total DNA from water samples to compare the microbial communities of two distinct coastal regions exposed to different anthropogenic pressures: the highly polluted Port of Genoa and the protected area of Montecristo Island in the Mediterranean Sea. Analysis of the metagenomes revealed significant differences in both microbial diversity and abundance between the two areas, reflecting their distinct ecological habitats and anthropogenic stress conditions. Our results indicate that the combination of next generation sequencing (NGS) technologies and bioinformatics tools presents a new approach to monitor the diversity and the ecological status of aquatic ecosystems. Integration of metagenomics into environmental monitoring campaigns should enable the impact of the anthropogenic pressure on microbial biodiversity in various ecosystems to be better assessed and also predicted.

## Introduction

Natural microbial diversity encompasses a broad spectrum of microorganisms (bacteria, fungi, viruses) that exert a strong influence on global processes such as the carbon, nitrogen and sulphur biogeochemical cycles. Quick responsiveness to environmental changes and the rapid reproductive capacity of microorganisms allow for changes in both the qualitative and quantitative composition of particular habitats. Indices of microbial diversity are considered a sensitive measure of the state of the environment and the health of a given habitat or ecosystem. Assessment of biodiversity, therefore, represents a keystone in understanding the complex processes within ecosystems and needs to be taken into account in decisions concerning environmental resource management and conservation priorities [1], [2].

In view of the intrinsic connection between environmental quality and human health [3], many data have been collected to characterize numerous sites exposed to pollution or, more generally, to environmental changes. The types of data recorded range from single analytical measurements (e.g. air temperature, solar radiation, concentration of chemicals) to integrated datasets including information about more complex ecological changes (e.g. fluctuations in biocoenoses, productivity, element cycling) [4], [5]. However, so far, relatively little attention has been given to a broad systematic assessment of microbial biodiversity, most likely because of the vast diversity of uncultured microbes [6] and the lack of appropriate methods that would allow studies to be performed in reasonable timescales and sampling resolutions.

Water quality assessment represents an important aspect of environmental monitoring but is commonly restricted to chemical monitoring, despite numerous studies indicating that biodiversity in marine ecosystems is consistently reduced because of anthropogenic contamination [7]. Alternative methods that better mirror these alterations are therefore needed in order to detect such highly complex changes. One technique that is particularly suited for this purpose is next generation sequencing (NGS), which enables new perspectives to be obtained through a metagenomics approach applicable to any environmental sample including water [8]. At the same time, metagenomic databases and analysis tools combined with modelling and GIS applications are becoming more widely available and represent an important new source of information for exploring the intrinsic complexity of microbial diversity in various habitats [9].

For a long time microbiological monitoring has been restricted to the detection of microbes affecting human health, excluding the majority of microbial species mainly because of technical limitations [10]. This has changed with the arrival of new sequencing technologies, and recent studies offer a more complete global view of microbial communities as indicators of environmental conditions [11], [12].

In the present study we demonstrate the applicability of metagenomic profiling to assessing the extent of anthropogenic impact on two different marine ecosystems, complementing traditional monitoring measurements such as chemical analyses. Water samples were collected from two coastal regions of the Mediterranean Sea, the Port of Genoa, in 2009 and the protected area around Montecristo Island in 2010, two areas distinguished by high and low anthropogenic impact, respectively. Sequencing of total community DNA allowed us to generate metagenomic profiles and compare the microbial diversity caused by the anthropogenic stress response in these two distinct coastal marine ecosystems.

## Methods

### Ethics Statement

For sampling in the port of Genoa, no specific permit was required for the described field studies since the area is not privately-owned or protected in any way. We confirm that the field studies did not involve endangered or protected species.

For the sampling in the protected area Montecristo Island, all necessary permits were obtained for the described field studies. A permission was requested to the regulatory body Ministero dell’Ambiente e della Tutela del Mare - Parco Nazionale Arcipelago Toscano, which authorized the sampling with the authorization n. L. 394191; D.P.R. 22/07/96.

### Sample Collection and Processing

**Figure 1:**
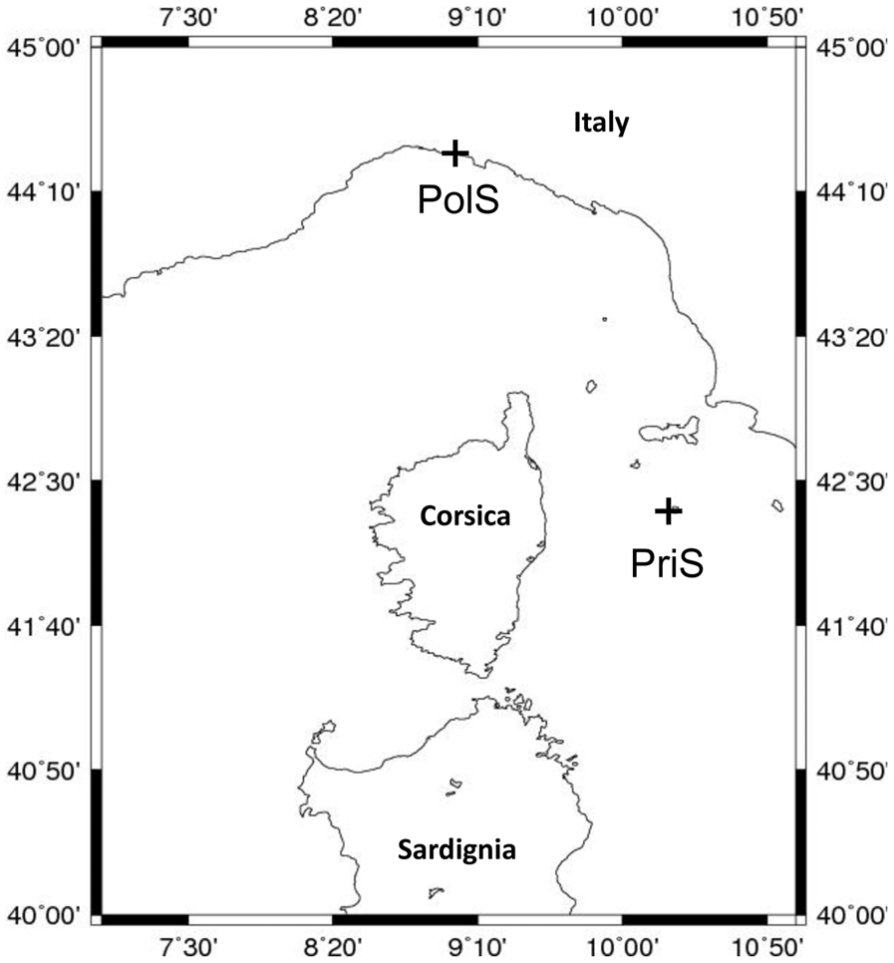
Sample site location. Geographic location of the sampling sites in the Mediterranean Sea. PolS – polluted site at Genoa Port; PriS – pristine site at the Montecristo Island.

Two coastal marine environments in the Mediterranean Sea were sampled using a sterile Ruttner sampler (Table 1, see Data S1). Sampling was performed on March 12 and September 18, 2009 at the Genoa Port site (PolS = polluted site) and on June 3 and August 26, 2010 at the Montecristo Island site (PriS = pristine site) (Fig. 1). During sampling, physical and chemical parameters (temperature, salinity/conductivity, pH, Chla fluorescence) were measured using a Hydrolab DSS probe. Water samples (20 liters) were collected twice (two replicates) at each of three different depths using sterile acid-washed Nalgene bottles. Samples were stored at −80°C unless used immediately for filtration and DNA extraction. Sediment samples (upper 3 cm) were collected at the same sites using a Grab Ekman Birge (Wildlife Supply, USA) and stored at −80°C. Statistically significant differences in physical and chemical parameters between samples were determined using the Kruskal-Wallis rank sum test.

**Table 1:** Background parameters collected for all samples.

Site	Polluted Site (Genoa Port 44° 24'203'', 8° 55' 470'')			Pristine Site (Montecristo Island 42°19' 44'', 10° 17' 29'')		
Date		Mar 12, 09	Sept 18, 09		Jun 3, 10	Aug 26, 10
Sample		PolS1	PolS2		PriS1	PriS2
	depth (m)			depth (m)		
Temperature (°C)	0	13.4±0.01	23.7±0.01	0	20.4±0.1	25.8±0.20
	4	13.5±0.01	23.7±0.01	7	19.9±0.1	25.6±0.02
	8	13.5±0.01	23.7±0.01	14	18.8±0.8	24.9±0.40
Salinity	0	36.3±0.08	36.1±0.01	0	36.6±0.02	38.7±0.03
	4	36.5±0.03	36.1±0.02	7	36.7±0.02	38.7±0.01
	8	36.5±0.02	36.2±0.02	14	36.8±0.09	38.7±0.05
Chla (µg Chlal^−1^)	0	0.57±0.09	0.67±0.02	0	0.04±0.01	0.18±0.04
	4	1.26±0.13	0.99±0.19	7	0.04±0.01	0.1±0.02
	8	1.25±0.05	0.84±0.16	14	0.37±0.10	0.1±0.02
TNB (10^6^ cells ml^−1^ )	0	1.47±0.20	0.71±0.07	0	0.70±0.07	0.40±0.04
	4	1.13±0.11	0.72±0.10	7	0.41±0.05	0.21±0.02
	8	0.58±0.08	0.70±0.07	14	0.62±0.06	0.33±0.04

Chla – chlorophyll a concentration, TNB – total number of bacteria, data: mean values ± SD of two replicates.

### Determination of Ultra-trace Elements in Marine Sediments and Sea Water

Each sediment sample (0.2 g) was analyzed by ICP-MS using an APEX system (Agilent, Santa Clara, CA, USA) following a microwave (Milestone ETHOS 900) assisted digestion [13]. For sea water analysis, a HMI (High Matrix Interface) system was added to an Agilent 7500 ICP-MS (Agilent, Santa Clara, CA, USA) instrument to allow ultra-trace metals to be analyzed without strong dilution.

### Extraction of Total Community DNA

Total community DNA was recovered from the shallow and homogeneous mixed water column by pooling these samples together. All samples collected from the PolS were pooled together (depth 0 to 8 m), while only samples from the upper layers (depth 0 to 7 m) of PriS were pooled together excluding the deepest samples (14 m). Prior to DNA extraction each sample of 20 liters was split into aliquots of approximately 250 ml to avoid clogging of the filters. Aliquots were then directly filtered on separate 0.22 µm pore size membrane filters (Millipore, GSWP04700). According to the Rapid Library Kit manufacturer's instructions (Roche), ∼500 ng of sampled DNA should be used for the preparation of pyrosequencing samples. Depending on the plankton density in the pooled samples a variable number of filters corresponding to different sample volumes were therefore needed in order to extract the desired amount (500 ng) of community DNA. As a consequence the total volume filtered for the March 12 and September 18, 2009 PolS samples was 5.05 and 5.625 liters, respectively, and volumes of 10 liters were filtered for each of the PriS sample. The filters were stored at –20°C. Before DNA extraction, the thawed filters were incubated and shaken (160 rpm) in 5 ml of 50 mM K_2_PO_4_ buffer overnight at 4°C for better recovery of cells. Subsequently, they were treated with 5 U µl^−1^ of lyticase (Sigma-Aldrich, USA), 4470 U µl^−1^ of lysozyme (Sigma-Aldrich,USA) and finally 2.5 µl of ß-mercaptoethanol (Sigma-Aldrich, USA). DNA was extracted using the DNeasy Blood and Tissue kit (Qiagen, UK) according to the manufacturer’s instructions. DNA was quantified on 1% agarose gel using a MassRuler™ High Range DNA Ladder (Fermentas, Canada).

### Determination of Cell Numbers

To determine bacterial abundance, samples fixed in 1% paraformaldehyde were sonicated for 5 min at 4°C (Bandelin Sonorex Digital 10P, 480W), stained with SybrGreen I (final concentration 5 µM) and analyzed by flow cytometry (BD LSR II, excitation by Solid State Sapphire L1 488 nm, band pass filters 530/30 nm). For quantification, ∼50×10^3^ particles of the internal standard were used (BD CountBright™ absolute counting beads, Ø 6 µm).

### Direct Pyrosequencing of Total Community DNA

Total community DNA was directly sequenced using the equipment and tools available at the Zürich Functional Genomics Centre according to the manufacturer's instructions: a GS Rapid Library Kit (Roche), Roche 454 Genome Sequencer FLX with the GS Titanium Sequencing Kit XLR70, GS Titanium PicoTiterPlate Kit (70×75) and gsRunProcessor from GS FLX SW v2.3. Two samples were run in parallel on one plate yielding 200–300 Mbp of sequence information per sample. The metagenomic data is available through the MG-RAST server (http://metagenomics.anl.gov/metagenomics.cgi?page=MetagenomeSelect) with ID numbers 4449589.3 (PolS1), 4449685.3 (PolS2), 4451102.3 (PriS1), 4451593.3 (PriS2).

### Sequence Read Processing and Annotation of Metagenomic Profiles

Raw reads were processed using the Rapid Analysis of Multiple Metagenomes with a Clustering and Annotation Pipeline (RAMMCAP [14]; http://weizhong-lab.ucsd.edu/rammcap) implemeted in CAMERA (http://camera.calit2.net/) and the MG-RAST server ([15]; http://metagenomics.anl.gov/). In RAMMCAP, exact read duplicates were removed using CD-HIT [16] and ribosomal sequences were predicted using HMMER3 [17]. tRNAs were predicted by tRNAscan-SE [18]. All RNA sequences were masked before further RAMMCAP analysis. ssuRNAs were classified into OTUs using the GreenGenes database and the NAST alignment tool ([19], [20]; http://greengenes.lbl.gov/cgi-bin/nph-index.cgi) applying a 70% sequence identity, 100 bp alignment length and 1.0e-05 e-value threshold.

In the MG-RAST analysis, the optional initial quality control (QC) filter was applied to the raw sequence data combined with their associated quality scores (FASTQ format) to remove duplicate and low quality reads. Organisms were classified in MG-RAST using the M5NR protein database (http://tools.metagenomics.anl.gov/m5nr/) applying an e-value threshold 1.0e-05. Functional annotation and classification relied on the KEGG Orthology ([21]; http://www.genome.jp/kegg/ko.html) or SEED subsystem ([22]; http://www.theseed.org/wiki/Home_of_the_SEED) databases applying an e-value threshold of 1.0e-05. Functional annotation with PFAM ([23]; http://pfam.sanger.ac.uk/) was performed using the CoMet server ([24]; http://comet.gobics.de). Annotation against the Clusters of Orthologous Groups (COG) database [25] was performed with RAMMCAP on all reads that passed the MG-RAST QC filter. Potential open reading frames (ORFs) were detected using ORF_finder (minimum ORF length: 40aa) and annotated against COG with RPS-BLAST [26] applying a hit e-value threshold ≤1.0e-05.

Gene family and category enrichment were analyzed on the metagenomic profiles using the ShotgunFunctionalizeR tool [27] implemented in R (http://www.r-project.org/). Functional annotation profiles obtained from RAMMCAP, MG-RAST or COMET were reformatted for ShotgunFunctionalizeR analysis using custom-generated Perl scripts (see Data S2). Prior to ShotgunFunctionalizeR analysis, all counts associated with an individual sample were normalized taking into account the number of total reads post MG-RAST QC.

## Results

### Environmental Parameters and Abundance of Bacteria

Samples were collected at two environmentally distinct sites in the Ligurian Sea during 2009 and 2010 (Figure 1). The Port of Genoa (polluted site, PolS) was chosen as the first site coinciding with an important container terminal of the Mediterranean Sea located in a highly urbanized and industrialized area [28]. In stark contrast to this, the second site (pristine site, PriS) is close to Montecristo Island, a protected natural reserve area in the Tuscan archipelago [29].

In order to determine general environmental parameters characterizing the two sites, samples were collected as two replicates at different depths within a column of homogeneously mixed water mass (Table 1). Salinity was slightly higher at PriS though this was not statistically significant (p>0.05). Average sample temperature reflected the expected seasonal variation with values being lowest in March, medium in June and highest in August and at the beginning of September. Chlorophyll a (Chla) concentration was significantly higher in PolS (median 0.86 µg Chla l^−1^) than in PriS (median 0.10 µg Chla l^−1^) (p<0.001), consistent with the expected high level of eutrophication at the polluted site. The same trend was observed for the total number of bacteria (TNB), which was significantly higher at PolS (median 0.71×10^6^ cells ml^−1^, p<0.01) than PriS (median 0.41×10^6^ cells ml^−1^). Taken together, the Chla and TNB values indicate a global shift in biodiversity towards planktonic organisms such as bacteria and algae consistent with a higher level of nutrients at the polluted site.

The concentration of trace metals in the water column as revealed by mass spectrometry did not differ between sites, with the exception of Mn, which was about three times higher at the polluted site (median in PolS 2.805 µg Mn l^−1^, median in PriS 0.955 µg Mn l^−1^, p = 0.048, see Data S3). Differences between the polluted and pristine sites were more pronounced in the sediment samples with several metals showing a statistically significant enrichment in PolS as compared to PriS: Cd (0.105 and 0.025 µg g^−1^, p = 0.007), Co (10.7 and 2.4 µg g^−1^, p = 0.045), Fe (21.1 and 9.1 µg g^−1^, p = 0.019) and Mn (880 and 202 µg g^−1^, p = 0.0005) (see Data S3).

### Direct Sequencing of PolS and PriS Samples

The global difference in biodiversity, already indicated by the Chla and TNB values, was examined in more detail using a metagenomic approach based on direct sequencing of water samples from the two sites. Samples were collected at different time points so any possible site-independent variations could also be captured, and total community DNA for sequencing was extracted from a total of four samples. Overall, about 2.5 million single reads (total about 10^9^ bps, average read length between 330 and 400 bps) were generated from the samples (Table 2) using standard pyrosequencing technology and protocols. Prior to further processing, the raw read data were subjected to a QC filter to remove lower quality and duplicate reads, the latter representing a phenomenon frequently observed during pyrosequencing [30], [31]. The filtering step removed between 12% and 33% of reads in each sample, with the highest value observed for PolS1 (Table 2). A complementary analysis using the CD-HIT clustering tool [16] revealed that within practically all clusters (clustered at the 96% identity level) individual reads started at exactly the same position. In a highly complex metagenome the probability of observing true duplicate sequences originating from exactly the same genome location is very low, even when one dominant operational taxonomic unit (OTU) is assumed [30]. The observed duplicate reads are therefore most likely to have been artificially generated during the sequencing reaction and the percentage of true natural duplicates should be very low [31]. Unique sequence reads passing the QC filtering step were then subjected to further analysis focusing on biodiversity and functional protein annotation.

**Table 2:** Overview of raw sequence read output, processing and annotation of metagenomic profiles.

	PolS1Plate #1	PolS2Plate #1	PriS1Plate #2	PriS2Plate #2	
**RAW Data**					
Total # of reads	571,744	568,630	671,764	693,149	
Total bp	194,960,711	186,243,134	269,776,240	245,234,759	
**MG-RAST Analysis**					
Total # of reads	571,744	568,630	671,764	693,149	
Total bp	194,960,711	186,243,134	269,776,240	245,234,759	
Average length (bp)	340±118	327±118	401±129	353±124	
Total # of reads post QC	383,131	439,385	523,347	596,911	
Total bp post QC	135,544,175	147,260,770	211,135,348	211,543,631	
Average length post QC (bp)	353±121	335±119	403±128	354±124	
Processed unique protein features	359,446	379,519	418,664	495,641	
Processed unique RNA features	61,724	73,720	102,658	121,498	
**RAMMCAP Analysis**					
# of reads 100%(CDHIT threshold)	563,654	563,247	659,813	689,527	
# of reads 96%(CDHIT threshold)	378,589	429,269	516,048	587,934	
# protein features analyzed	803,461	812,495	1,413,570	1,420,763	
# of COG hits (e-value >1.0e-05)	139,603	140,392	254,215	227,479	
# of COG families	3452	3248	3248	3342	
**Phylogenetic marker**s					
# of ssu rRNAs (RAMMCAP)	938	710	759	987	
# of ssu rRNAs after NAST	800	623	668	854	
# of OTUs (GreenGenes)	182	130	180	258	
**MG-RAST Functional Annotation**					
# of KEGG Orthology entries	224	213	223	231	
# of SEED subsystem level 2	424	423	422	426	
# of SEED subsystem level 3	757	746	738	756	
**COMET Functional Annotation**					
# of PFAM families	6651	6410	6822	7167	

### Biodiversity in PolS and PriS Samples

**Figure 2:**
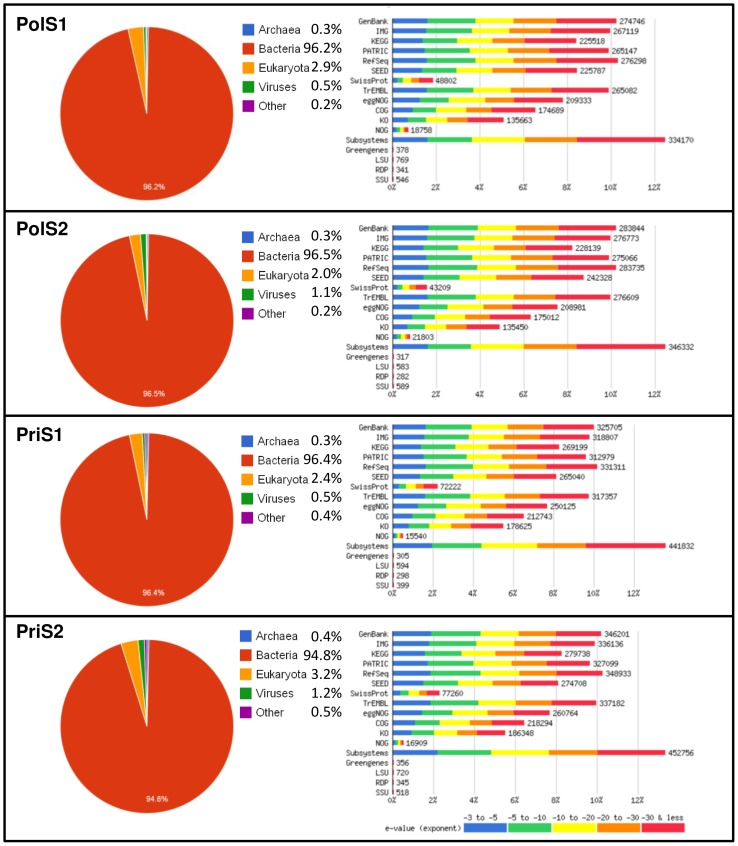
Taxonomic annotation by MG-RAST. Pie charts summarizing the combined taxonomic domain information obtained through annotation against the M5NR database. Bar-chart diagrams on the right indicate extent and quality (e-value distribution) of the annotation from each individual database. Similarities below the 1.0.e-05 e-value threshold, generally less than 20% for each individual database, are coloured blue.

Domain distributions in the four samples, determined using the MG-RAST M5NR database, showed the expected dominance of Bacteria (>94%), a small fraction of Eukaryotes (2–3%) and generally less than 1% of Archaea, viruses and other unclassified organisms (Fig. 2), values generally observed in coastal sea samples [32], [33].

**Figure 3:**
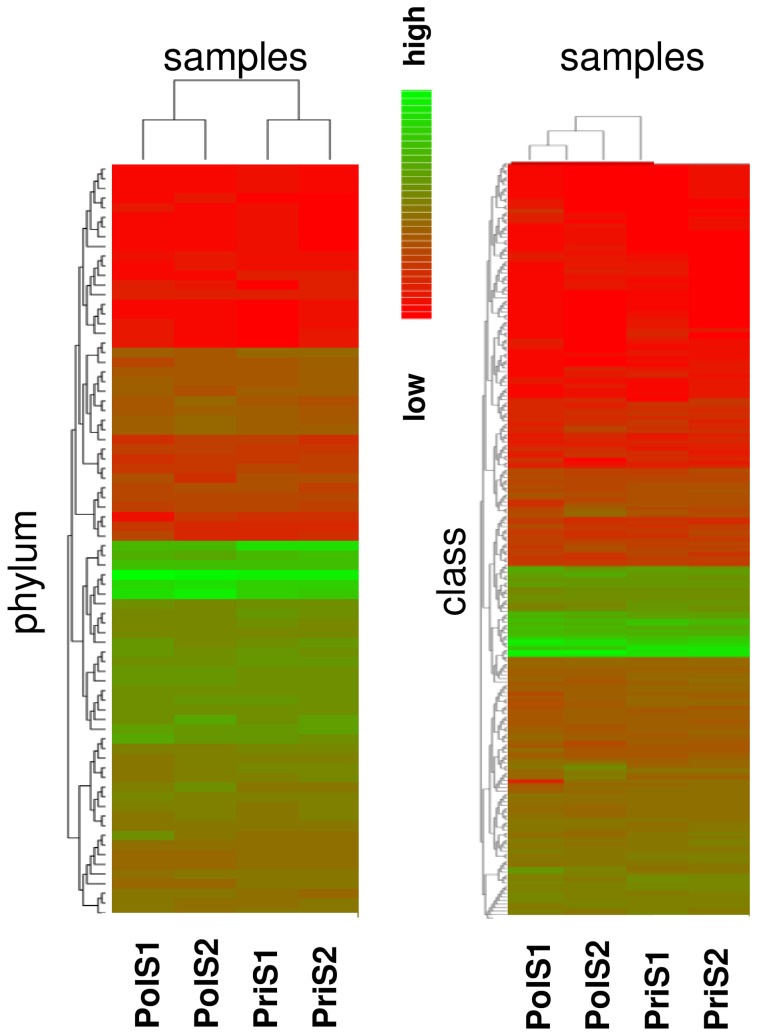
Phylogenetic clustering. MG-RAST heatmaps representing the phylogenetic diversity of the four samples at the phylum (left) or class (right) level. Differences between PolS1 and PolS2 are more pronounced than between PriS1/PriS2 sample pairs. Red and green colours indicate low and high abundance, respectively.

**Figure 4:**
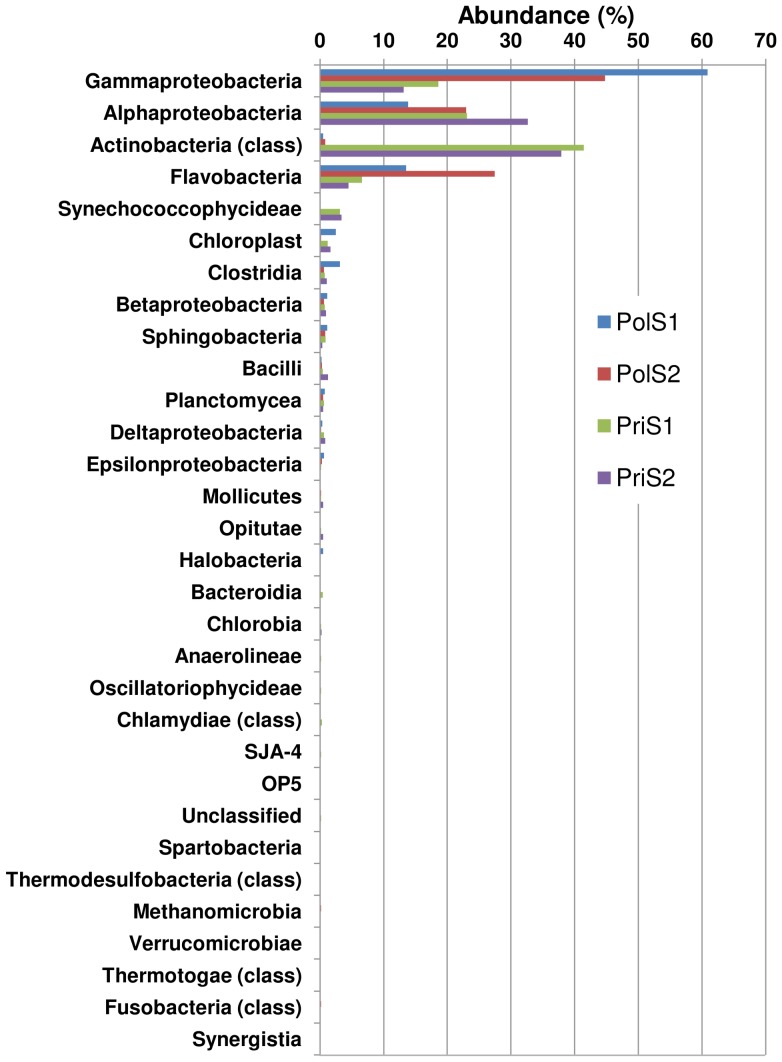
Phylogenetic community diversity. Diversity represented at the class level as determined using GreenGenes. Values reported represent the percentage fraction present in each sample. Gammabacteria are enriched in the polluted samples PolS1/PolS2 whereas Actinobacteria dominate in the pristine samples PriS1/PriS2.

**Figure 5:**
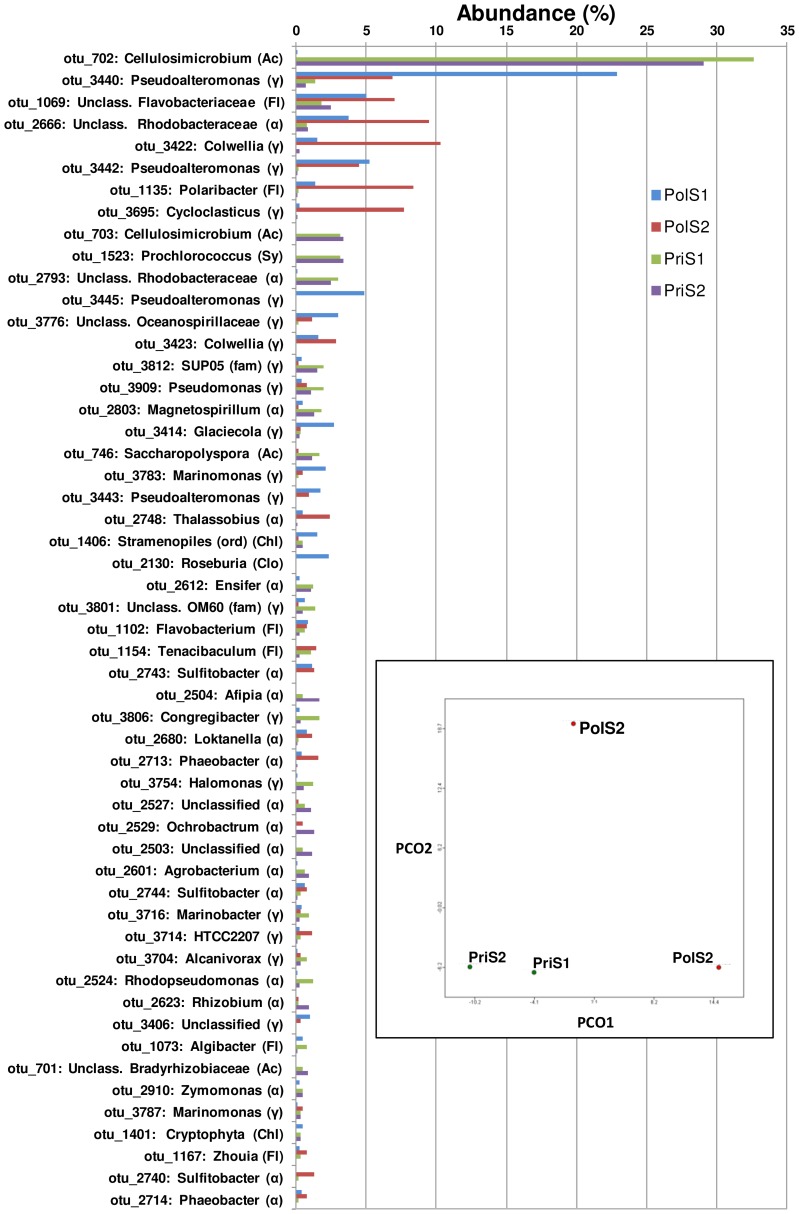
OTU distribution based on ssu rRNAs. OTU distribution as determined by GreenGenes. Only the most frequently encountered OTUs representing about 50% of the total are shown (complete list in Data S4). Clear differences are apparent not only between the two sample classes but also between the two polluted samples PolS1 (blue bars) and PolS2 (red bars). Labels indicate the OTU class: α – Alphaproteobacteria, β - Betaproteobacteria, γ - Gammaproteobacteria, Ac – Actinobacteria, Ba – Bacteroidetes (Flavobacteria), Cy – Cyanobacteria, FL – Flavobacteria, Sy – Synechococcophycideae, Chl - Chloroplast, Clo - Clostridia. **(Insert)** Principal component analysis of sample diversity using the MG-RAST M5NR protein classification. PriS1 and PriS2 nearly coincide, in contrast to PolS1 and PolS2, which are well separated.

Annotation quality was consistently high across all M5NR database sources with more than 80% of similarities falling below the e-value cutoff value of 1.0e-05 subsequently applied during functional characterization of the metagenomic profiles. A more detailed comparison of organism abundance at the phylum and class levels correctly grouped the samples according to their origin, with some intra-group variations recognizable especially between the polluted samples PolS1 and PolS2 (Fig. 3). Both inter- and intra-group differences became more pronounced when ssuRNAs were classified by GreenGenes at the class (Fig. 4) or OTU (Fig. 5) level. Gammaproteobacteria, Alphaproteobacteria, Flavobacteria and Actinobacteria dominated the class distribution with Actinobacteria being practically absent in the polluted samples. At the OTU level more subtle differences became apparent (Fig. 5, see Data S4), being particularly pronounced when the two polluted samples were compared. Principal component analysis on the MG-RAST global organism classification profile confirmed this result showing the polluted samples to be well separated, quite distinct from the pristine samples, which nearly coincide (Fig. 5). Consistent with this finding, overlap between the latter samples at the OTU level was considerably greater than between the polluted samples (Fig. 5).

### Functional Annotation of Predicted ORF Fragments and Comparison of Metagenomic Profiles

Predicted protein features were annotated using a panel of complementary tools: RAMMCAP for COG protein families [25], [14], COMET for PFAM family profiles [23], [24], MG-RAST [15] for KEGG pathways [34] and SEED subsystems [22]. For consistency the RAMMCAP and COMET annotations were performed on reads that passed the initial MG-RAST QC filter. In all cases, to avoid bias from lower quality spurious hits, an e-value threshold of 1.0e-05 was applied when the results were exported or further processed. Extrapolating from the results obtained during the COG analysis, about 40% of predicted protein features were successfully annotated, with only a minor fraction of reads (<1%) containing two distinct annotated protein features. The annotation results obtained for the individual metagenomic profiles were then combined into a format suitable for analysis with ShotgunFunctionalizeR [27] in order to detect significant differences between the two groups of samples (polluted and pristine) and also between individual samples within each group. To correct for different global sample sizes the individual counts were normalized with respect to the total number of predicted protein features prior to ShotgunFunctionalizeR analysis.

**Figure 6:**
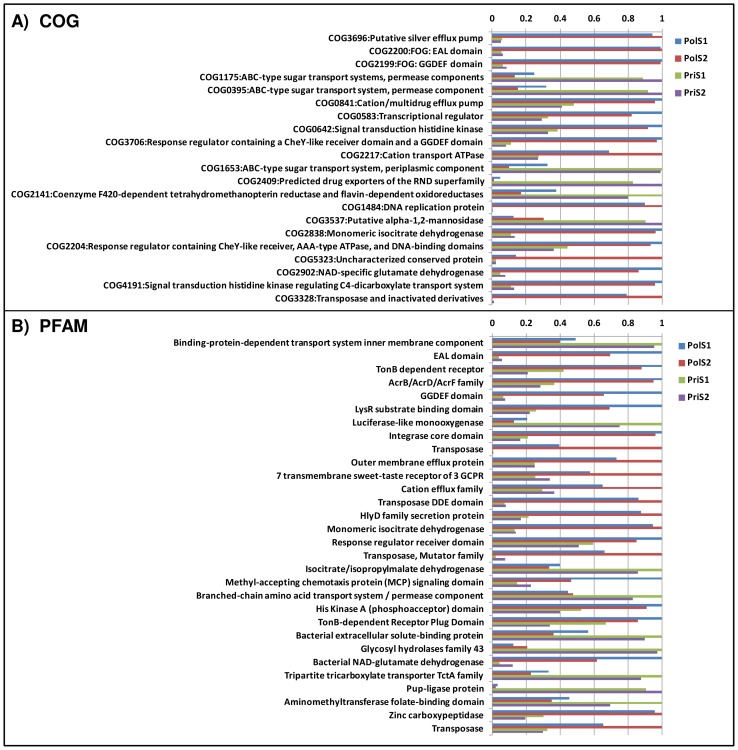
Functional protein annotation of metagenomic profiles. A . RAMMCAP COG annotation. Only the 20 COG families with the most significant differences (ShotgunFunctionalizeR) between pairs of polluted and pristine samples are shown (complete list in Data S5). **B**. Top 30 PFAM families showing the most significant differences (ShotgunFunctionalizeR) between the polluted and pristine samples in the COMET annotation profiles (complete list available in Data S5). Values shown represent the relative abundance of each sample with respect to the sample with the highest abundance (normalized to 1).

The enrichment analysis highlighted a large number of COG families with significant differences between the two sample groups (Fig. 6a, see Data S5). In particular, ionic transport systems (silver efflux pump, cation transport ATPase), functions related to recombination (transposase) and several COG families implicated in signalling processes (e.g. EAL domain, GGDEF domain, signal transducing histidine kinase, CheY-like receiver) were present predominantly in the polluted samples. In contrast, the pristine samples showed a much more pronounced prevalence of transport functions such as ABC-type sugar transport systems and functions related to coenzyme 420 (Fig. 6a). PFAM analysis essentially mirrored the distribution from COG e.g. with luciferase-like monooxygenase, a coenzyme 420 dependent activity and Pup-protein ligase dominating in the PriS samples. Similarly, among the highest scoring PFAM families, several signal transduction functions (EAL, TonB, GGDEF) and processes related to recombination such as Integrase or Transposase were more frequent at the polluted site (Fig. 6b).

**Figure 7:**
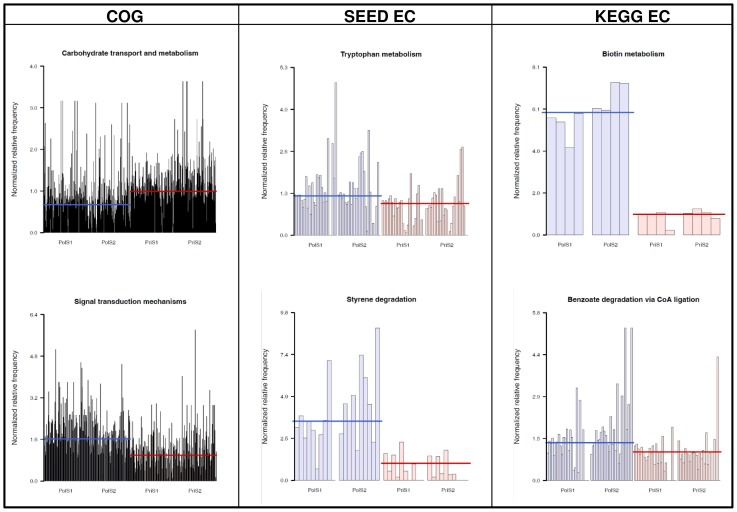
Functional annotation at the pathway and category level. Examples of whole categories (COG) or pathways (KEGG, SEED) showing significant differences between the two sample groups as determined by ShotgunFunctionalizeR. A complete list is available in Data S6.

**Figure 8:**
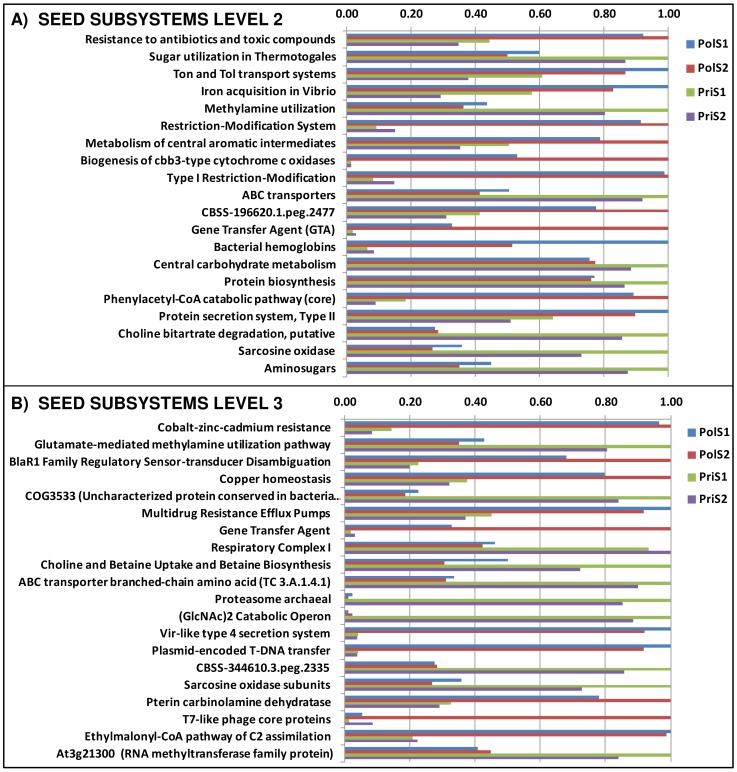
Functional annotation using the SEED subsytem definition. Comparison of metagenomic profiles at **A**. the SEED subsystem level 2 or **B**. at the SEED subsystem level 3. Analysis was performed using ShotgunFunctionalizeR with SEED annotation results from MG-RAST. Only a selection of subsystems showing the greatest differences between the two sample groups is shown. The complete list is available in Data S5.

Because ShotgunFunctionalizeR also allows groups of functionally connected genes, e.g. whole pathways, to be examined concurrently, the analysis was extended to COG gene categories and enzymatic pathways. Several COG categories differed significantly between the sample groups (see Data S6) with “Signal transduction” and “Carbohydrate transport and metabolism” (Fig. 7) appearing at the top of the list, in agreement with the COG family-focused analysis. Many differences were also observed (see Data S6) for metabolic pathways (mapped through the EC numbers from the MG-RAST KEGG and SEED annotation), including the prevalence of several degradation pathways such as “Styrene degradation” and “Benzoate degradation via CoA ligation” in the polluted sample group. MG-RAST also allows metagenomic profiles to be analyzed at different hierarchical levels of SEED subsystem classification. The annotation results from the SEED database were therefore re-processed at two different levels of complexity, the subsystem level 2 (Fig. 8a) and the subsystem level 3 (Fig. 8b). The results closely matched the previous findings from the COG and PFAM analysis indicating signal transduction, transport and restriction functions among the subsystems with the greatest differences. In addition, several subsystems in the polluted sample group involving heavy metals (“cobalt-zinc-cadmium resistance”, “copper-homeostasis”) or connected to compound resistance (“Multidrug Resistance efflux pump”, “Resistance to antibiotic and toxic compounds”) were significantly enriched, reflecting the more hostile harsh environmental conditions.

The biodiversity distribution within the four metagenomic profiles had indicated a substantial difference between the two polluted samples, in particular at the OTU level. Therefore, functional protein annotation was also compared separately within each group, again using ShotgunFunctionalizeR. For the two pristine samples very few COG families showed a significant difference (Benjamini-Hochberg (BH) adjusted p-value <1.0E-05), confirming their high similarity. In contrast, the abundances of many more COGs differed between the two polluted samples (Data S6). However, importantly, only a small number of these COG families had also been found in the between-groups ShotgunFunctionalzie comparison (see Data S7). A small bias due to the more heterogeneous composition of the polluted samples can therefore not be excluded; nevertheless, the major conclusions from the group-wise analysis should be considered valid.

## Discussion

Present practice in environmental monitoring lacks tools that are adequate for detecting and analyzing the impact of complex factors on aquatic and terrestrial ecosystems. In particular, monitoring the health of aquatic environments typically focuses on chemical pollutants [37], excess of nutrients leading to eutrophication, and biological indicators for pathogen contamination [38], [39]. Although such data certainly provide critical parameters describing the health of the environment, more subtle (and earlier) indicators of alterations caused by multiple stress factors might be missed [40]. Since in general all organisms within a particular ecosystem will be affected and changes might be relatively small, a more global, but at the same time more detailed, view of community composition could provide important information. Methods such as the metagenomic analysis of water samples performed in the present study provide such a global view, thus complementing the more traditional approaches.

In the present study two coastal marine systems exposed to rather different anthropogenic pressures have been examined: the Port of Genoa, located in a heavily urbanized and industrialized area, and the uninhabited Montecristo Island resource. In both cases, samples were collected before and at the end of the summer period of 2009 (Port of Genoa) or 2010 (Montecristo). A more synchronized schedule might have been preferred at first glance, but one aim of the present study was to gain insight into how much sample variation should be expected when sampling a large complex ecosystem. In routine monitoring practice, water samples would in general be taken only from “suspected” sites and not necessarily always at the same time point during the year. Knowledge about the potential global variations in the polluted samples will be important for comparing them optimally to a “healthy” reference sample (or reference profile). We have therefore chosen, as a first test case, to examine four water samples collected at different time points from two environmentally very distinct sites located in distant areas (several hundred kilometres apart) of the Mediterranean coastal sea.

Analysis of the metagenomic profiles proved consistent with the expected sample characteristics. The functional protein annotation recapitulated the results from phylogenetic analysis well, showing clear differences between the two sample groups exposed to distinct anthropogenic pressures. In the pristine sample site, the high abundance of Actinobacteria and the associated enzymatic functions and pathways detected are consistent with an environment containing oligotrophic organisms adapted to low levels of nutrients and characterized by a high potential for utilizing low concentrations of organic substrates through energy-efficient ABC transporter systems [41]. Expansion of the ABC transporters is also consistent with an environment in which nutrients are scarce and uptake needs to be optimized as much as possible. In addition, several functional traits characteristic of Actinobacteria [35], [36] were overrepresented in samples collected from the pristine site e.g. luciferase-like monooxygenase, a coenzyme 420 dependent activity, and Pup-protein ligase, consistent with the observed frequencies of phylogenetic makers (rRNA genes) (Figs. 4 and 6).

In contrast, polluted environments are generally characterized by high levels of numerous stress factors and high level of nutrients.

Organisms adapted to such environments are in general copiotrophs, e.g. Gammaproteobacteria, which are the “feast and famine” strategists optimizing rapid growth in the presence of labile nutrients. Reflecting this characteristic, the microbial community at the polluted site had significantly higher levels of signal transduction regulator proteins involved in a variety of cellular responses to environmental stimuli [41]. Enrichment of the functional community profile with efflux pumps and cation transport systems indicates the necessity for increased protection against toxic compounds such as metal ions, consistent with the high concentrations of several metals in the sediment (Data S2).The polluted samples were also enriched in functions related to cell motility, intracellular trafficking and secretion, features linked to the tendency of copiotrophs to gain access to nutrient-enriched patches in open water environments. Over-representation of genes belonging to signal transduction functions (EAL, TonB, GGDEF) and processes related to recombination e.g. Integrase or Transposase suggest regulation of gene expression in response to external and internal stimuli and increased horizontal gene transfer. Together, these functional traits facilitate the adaptation of the microorganisms to an environment characterized by highly heterogeneous, variable and unfamiliar stimuli from anthropogenic sources.

Despite being collected at different seasonal time points during the year, the metagenomic profiles from the two pristine samples closely resembled each other. Generation of a “healthy” reference profile therefore seems feasible and further refinement through incorporation of additional samples should allow the profile to be consolidated. In contrast, the metagenomic profiles of the two polluted samples exhibited much larger variation, probably reflecting fluctuations of the harsher environmental conditions caused by quantitative and qualitative variations of pollutants. Consequently, a much larger sampling study will be required to identify robust generally-applicable indicators of pollution. Importantly, in order to extend the applicability of metagenomic monitoring to potentially much less polluted samples (and allow negative shifts in community profiles to be recognized early), a large number of samples with a broad distribution of pollution levels will have to be examined.

Compared to more traditional methods, PCR-based direct sequencing of total community DNA mostly avoids several of the methodological problems that potentially introduce various biases [42], [43]. For example, use of a single set of universal rRNA gene primers has been reported to miss up to 50% of the total microbial species richness [44], [45]. In addition, specific phylogenetic markers such as rRNA genes do not provide information regarding the spectrum and prevalence of functional properties within a community, i.e. properties associated with protein-coding genes. The presence of anthropogenic pressures will modulate biodiversity and also, consequently, the overall prevalence of protein-coding genes belonging to particular functional classes. The metagenomic approach does enable global profiles of predicted protein-coding genes to be compared among different communities and can therefore reveal characteristic differences caused by distinct environmental conditions. However, 454 pyrosequencing data in particular have to be critically examined to detect possible systematic artefacts. In our hands, up to 33% of reads might actually be artificial replicates, a fraction similar to what has been reported previously [30], [31]. Fortunately, new tools are becoming available for detecting and estimating these sources of error, thus enabling artificial replicates to be removed efficiently [46], [47] and allowing for correct interpretation of metagenomic data.

Although it is still at an early stage, metagenomic analysis of total community DNA using direct sequencing without cloning and with long sequence fragments (∼400 bp or more) opens up new perspectives in environmental monitoring by providing a relatively simple, robust and reproducible approach to studying samples with unknown diversity and exposed to unknown anthropogenic pressures. Until very recently 454 pyrosequencing had the advantage to produce longer average read length compared to the rather short reads (<100 bp) obtained by Illumina, the main alternative and cheaper technology. Since the average gene length in bacteria is about 1 kB [48], the former technology was chosen for the present study, however at a considerably higher cost. Rapid development and improvements of other technologies have closed this gap in the meanwhile increasing both average read length and the amount of data generated. In a recent direct comparison of the two technologies (Roche 454 FLX Titanium vs Illumina Genome Analyzer II) on a real metagenomic sample, Illumina was judged comparable, if not higher performance, to the 454 system [49]. Certainly the lowering cost will pave the way for a broader and more intense application of metagenomics and its complementary metatranscriptomics [50], in order to obtain more detailed and broad understanding of the potential changes induced in marine microbial communities under altered environmental conditions. In this perspective our results represent only a first step and more systematic broad studies will be needed to robust monitoring practises based on metagenomic profiles.

Finally, it should not be neglected that microorganisms are known to make up the bulk of the biota in both natural and managed ecosystems. Proposals to restore the function and integrity of ecosystems are increasingly being put forward, and any initiative would be incomplete if considerations regarding the complexity and integrity of the underlying microbial systems are not included.

## Supporting Information

Data S1 **Minimal Information about Metagenomics Sequences (MIMS).**
(XLS)

Data S2 **Description of the analysis workflow together with a collection of custom-written Perl scripts and example data files.**
(ZIP)

Data S3 **Table describing sediment metal concentrations.**
(XLS)

Data S4 **Complete GreenGenes OTU classification table.**
(XLS)

Data S5 **Complete table describing sample differences by functional annotation against COG, Pfam, SEED and KEGG (ShotgunFunctionalizeR analysis).**
(XLS)

Data S6 **Complete table describing sample differences by pathway annotation against COG, SEED and KEGG (ShotgunFunctionalizeR analysis).**
(XLS)

Data S7 **ShotgunFunctionalizeR analysis comparing the COG functional annotation of two pristine or the two polluted samples.**
(XLS)
