# Supplementary material for: Phylogenetic and Functional Metagenomic Profiling for Assessing Microbial Biodiversity in Environmental Monitoring
Source: PLoS One. 2012 Aug 27;7(8):e43630. doi: 10.1371/journal.pone.0043630 (PMC3428334; doi:10.1371/journal.pone.0043630)
Supplement: Data S2 — Description of the analysis workflow together with a collection of custom-written Perl scripts and example data files. (ZIP) [file pone.0043630.s002.zip › Data_processing_workflow.doc]

**Supplementary Data 2: Analysis workflow**

**RAMMCAP analysis:**

After installation of the RAMMCAP analysis package (<http://weizhong-lab.ucsd.edu/rammcap>) the four samples were submitted separately to the RAMMCAP pipeline using the run_rammcap.csh shell script (a slightly modified version of the original RAMMCAP example script):
 ./run_rammcap.csh FASTAFILE
where FASTAFILE represents a FASTA format file containing the reads for one of the samples.

The script applies the following analysis steps:

Step 0): Pre-filter step to remove 100% exact duplicate reads using program cd-hit-454 (Ref 1). Parameters applied were –c 1.0 (100% sequence identity), -b 1 (bandwth, default value), -aL 1.0 (100% of alignment coverage for longest sequence), -aS 1.0 (100% of alignment coverage for longest sequence

Step 1,2) rRNA and tRNA identification (default parameters as present in the original RAMMCAP example script). Result is one file containing the reads with rRNA and tRNA sequences masked and a second file containing the detected 16S RNA sequence fragments.

Step 3) ORF identification: The output file from step 2 (FASTA file with rRNA and tRNA sequences masked) is analyzed for the presence of ORFs. Parameters applied were –l 40 (minimum ORF length 40aa), -L 40 (minimum ORF length between stop codons), -t 11 (translation table bacteria)

Step 4) Split of ORFs identified in step 3) into multiple files for step 5)

Step 5) COG annotation of ORFs identified in step 3).

The final output file orf-COG-annotation.txt contains the list of COG hits for each of the samples analyzed. Results from the four samples were then combined using the cog-summary.pl perl script described below.

**Greengenes classification:**

For each of the four samples, the 16S RNA sequences obtained from step 1) of the RAMMCAP analysis were submitted to the NAST tool (<http://greengenes.lbl.gov/cgi-bin/nph-NAST_align.cgi>) in blocks of less than 500 sequences as recommended by the server. Parameters used were 70% minimum identity and 100 minimum length. For each block of sequences the output file obtained from NAST was saved and then combined into one file. This combined file was then, for each sample separately, submitted to the GreenGenes classification against multiple taxonomies (<http://greengenes.lbl.gov/cgi-bin/nph-classify.cgi>). The output of each analysis (containing classification results at the kingdom, phylum, class, order, family, genus, species, otu level) was saved as a tab-separated file.

classifications_greengenes_PolS1.txt

classifications_greengenes_PolS2.txt

classifications_greengenes_PriS1.txt

classifications_greengenes_PriS2.txt

Results for the four samples were then combined using the perl script greengenes_summary.pl. The script reads the four classification results files and generates four output files containing the classification results for the four samples at the Greengenes order, class, phylum and OTU level, respectively:

greengenes_classification_summary_class_level.txt, greengenes_classification_summary_order_level.txt, greengenes_classification_summary_phylum_level.txt, greengenes_classification_summary_otu_level.txt).

The data shown in Figures 4 and 5 represent the combined results for the class (Fig. 4) and OTU level (Fig. 5) following some reformatting in EXCEL (see also Data S4).

**COG functional annotation:**

The COG annotation files obtained for the four samples from the RAMMCAP analysis step 5 were combined into one file for further ShotgunFunctionalizeR analysis using the perl script cog-summary.pl. The script reads a small file providing sample names and the number of post QC reads for normalization
 ./cog-sumamry.pl samplenames.txt
and outputs a combined summary file (COG_shotgun.txt) that is then analyzed by ShotgunFunctionalizeR (see below).

**MG-RAST analysis: (http://metagenomics.anl.gov/metagenomics.cgi?page=Home)**

**Processing of raw data:** FASTQ format raw data files for the four samples were uploaded to the MG-RAST server and subjected to the MG-RAST QC pipeline. Quality filtering was selected as option as well as dereplication in order to remove both low quality reads (read length, number of ambiguous bases and quality values) and artificially duplicated reads (1) (dereplication: reads with identical first 50 base-pairs are binned and only one copy per bin is retained).

**Functional annotation:** Functional annotation against different data sources were extracted from the MG-RAST analysis results using the tools available on the MG-RAST website (<http://metagenomics.anl.gov/metagenomics.cgi?page=Analysis>) under the Functional Abundance (Hierarchical classification) option and choosing the desired annotation source (default parameters). Combined annotation results for the four samples against the SEED subsystems or the KEGG KO classification were saved as a tab-separated file (e.g. mydata_seed.tsv and mydata_ko.tsv). Annotation results at various SEED levels (level 2, level 3, functional level, functional level EC numbers) or the KEGG KO (plus KO with EC numbers) classification were than extracted and prepared for ShotgunFunctionalizeR analysis using two perl scripts (reformat_functions_seed.pl and reformat_functions_ko.pl ). Both scripts read two input files: one containing, for each sample, the MG-RAST sample ID, a sample name tag and the MG-RAST # of features after QC (for normalization) and the second file containing the saved MG-RAST functional analysis results. The script reformat_functions_seed.pl generates eight output files suitable as input for the ShotgunFunctionalizeR analysis: a data file and an annotation file for each of the following SEED levels: level 2, level 3, functional level, functional level EC numbers. The annotation files were necessary since, in contrast to COG which is explicitly known to ShotgunFunctionalizeR, the SEED subsystems are not known by ShotgunFunctionalizeR and require explicit annotation. The same is true for KEGG KO and the script generates four output files, the two data files (KO and KO with EC numbers) and the corresponding annotation files.

Running for example the reformat_functions_seed.pl script on a saved MG-RAST analysis file called mydata_seed.tsv
 ./reformat_functions_seed.pl mydata.samples.txt mydata_seed.tsv
the following files would be generated:

mydata_seed.tsv.list.shotgun.seed.level2.annotation.txt
mydata_seed.tsv.list.shotgun.seed.level2.txt

mydata_seed.tsv.list.shotgun.seed.level3.annotation.txt
mydata_seed.tsv.list.shotgun.seed.level3.txt

mydata_seed.tsv.list.shotgun.seed.funclevel.annotation.txt
mydata.tsv.list.shotgun.seed.funclevel.txt

mydata_seed.tsv.list.shotgun.seed.ec.annotation.txt
mydata_seed.tsv.list.shotgun.seed.ec.txt

Likewise, running the script ./reformat_functions_ko.pl
 ./reformat_functions_ko.pl mydata.samples.txt mydata_ko.tsv
the following files would be generated:

mydata_ko.tsv.list.shotgun.ko.annotation.txt
mydata_ko.tsv.list.shotgun.ko.txt

mydata_ko.tsv.list.shotgun.ko.ec.annotation.txt
mydata_ko.tsv.list.shotgun.ko.ec.txt

**PFAM functional annotation:** For PFAM, not available in MG-RAST, the CoMet server (<http://comet.gobics.de/>) was used. For each of the four samples, a FASTA format file containing the raw reads that passed the MG-RAST QC filter were downloaded from the MG-RAST server and submitted to CoMet. The CoMet results were saved into a tab-seperated file and the four results files were than combined using the perl script pfam_summary.pl. The script reads a text file containing for each sample a name tag and the # of reads post MG-RAST QC (for normalization) and generates two output files for ShotgunFunctionalizeR analysis: one containing the data (pfam_shotgun.txt) and containing the annotation data (pfam_shotgun.annotation.txt), similar to the scripts described above for SEED and KO.

**ShotgunFunctionalizeR analysis**: Results obtained for the two sample groups (PolS1/PolS2 and PriS1/PriS2) were compared using ShotgunFunctionalizeR ( <http://shotgun.math.chalmers.se/>) implemented in R (<http://www.r-project.org/>). For each comparison (e.g. SEDD level 3) the files generated with the corresponding perl scripts described above were read into R and analyzed with the ShotgunFunctionalizeR function testGeneFamilies.dircomp (individual gene families) or with the function testGeneCategories.dircomp (whole pathways containing various gene families). The R code that was used to generate the ShotgunFunctionalizeR results is given in ShotgunFunctionalizeR_R_code.txt

Results from ShotgunFunctionalizeR (output files called ...stat.txt in the R code) were then read into EXCEL and manipulated in order to generate the plots present in figures 6 and 8. Figure 7 was generated by the TrendPlot function in ShotgunFunctionalizeR.

**Content of zip-file:**

```
Data_processing_workflow.doc

greengenes_summary.pl			: perl script to combine Greengenes results
						  and extract classification levels
classifications_greengenes_PolS1.txt	: Greengenes results for PolS1
classifications_greengenes_PolS2.txt	: Greengenes results for PolS2
classifications_greengenes_PriS1.txt	: Greengenes results for PriS1
classifications_greengenes_PriS2.txt	: Greengenes results for PriS2
greengenes_classification_summary_class_level.txt  : output from greengenes_summary.pl
greengenes_classification_summary_order_level.txt  : output from greengenes_summary.pl
greengenes_classification_summary_otu_level.txt    : output from greengenes_summary.pl
greengenes_classification_summary_phylum_level.txt : output from greengenes_summary.pl


cog-summary.pl				: Perl script to combine COG results
COG_shotgun.txt				: Input file for ShotgunFunctionalizeR
cog.shotgun.stat.txt			: Output file from ShotgunFunctionalizeR
cog_pathways.shotgun.stat.txt		: Output file from ShotgunFunctionalizeR

reformat_functions_ko.pl	: perl script to reformat MG-RAST KO results
samplenames_ko.txt  	: example samplename file for reformat_functions_ko.pl
functions_ko_e5.tsv		: MG-RAST KO results
functions_ko_e5.tsv.list.shotgun.ko.txt	: output file for  ShotgunFunctionalizeR
functions_ko_e5.tsv.list.shotgun.ko.annotation.txt : output file for
      ShotgunFunctionalizeR
functions_ko_e5.tsv.list.shotgun.ko.ec.txt  : output file for  ShotgunFunctionalizeR
functions_ko_e5.tsv.list.shotgun.ko.ec.annotation.txt    : output file for
             ShotgunFunctionalizeR
functions_ko.shotgun.stat.txt		: Output file from ShotgunFunctionalizeR
functions_ko_ec.shotgun.stat.txt		: Output file from ShotgunFunctionalizeR
functions_ko_ec_pathways.shotgun.stat.txt : Output file from ShotgunFunctionalizeR


reformat_functions_seed.pl	: Perl script to extract MG-RAST SEED level results
functions_subsystems_e5.tsv.list.shotgun.seed.level2.txt
						: Input file for ShotgunFunctionalizeR
functions_subsystems_e5.tsv.list.shotgun.seed.level2.annotation.txt
						: Input file for ShotgunFunctionalizeR
functions_subsystems_e5.tsv.list.shotgun.seed.level3.txt
						: Input file for ShotgunFunctionalizeR
functions_subsystems_e5.tsv.list.shotgun.seed.level3.annotation.txt
						: Input file for ShotgunFunctionalizeR
functions_subsystems_e5.tsv.list.shotgun.seed.funclevel.txt
						: Input file for ShotgunFunctionalizeR
functions_subsystems_e5.tsv.list.shotgun.seed.funclevel.annotation.txt
						: Input file for ShotgunFunctionalizeR
functions_subsystems_e5.tsv.list.shotgun.seed.ec.txt
						: Input file for ShotgunFunctionalizeR
functions_subsystems_e5.tsv.list.shotgun.seed.ec.annotation.txt

functions_seed_level2.shotgun.stat.txt      : output file from  ShotgunFunctionalizeR
functions_seed_level3.shotgun.stat.txt      : output file from  ShotgunFunctionalizeR
functions_seed_funclevel.shotgun.stat.txt   : output file from  ShotgunFunctionalizeR
functions_seed_ec.shotgun.stat.txt          : output file from  ShotgunFunctionalizeR
functions_seed_ec_pathways.shotgun.stat.txt : output file from  ShotgunFunctionalizeR

pfam_summary.pl				: Perl script to combine PFAM results
pfam_shotgun.txt				: Input file for ShotgunFunctionalizeR
pfam_shotgun.annotation.txt		: Input file for ShotgunFunctionalizeR
pfam.shotgun.stat.txt			: Output file from  ShotgunFunctionalizeR

run_rammcap.csh 				: example shell script for RAMMCAP


ShotgunFunctionalizeR_R_code.txt		: R code for ShotgunFunctionalizeR
```
